# Current understanding of metal ions in the pathogenesis of Alzheimer’s disease

**DOI:** 10.1186/s40035-020-00189-z

**Published:** 2020-04-03

**Authors:** Lu Wang, Ya-Ling Yin, Xin-Zi Liu, Peng Shen, Yan-Ge Zheng, Xin-Rui Lan, Cheng-Biao Lu, Jian-Zhi Wang

**Affiliations:** 1 Key Laboratory of Brain Research of Henan Province, Sino-UK Joint Laboratory of Brain Function and Injury of Henan Province, Department of Physiology and Neurobiology, Xinxiang Medical University, Xinxiang, 453003 China; 2 Department of Pathophysiology, School of Basic Medicine, Key Laboratory of Ministry of Education of China for Neurological Disorders, Tongji Medical College, Huazhong University of Science and Technology, Wuhan, 430030 China

**Keywords:** Metal ions, Alzheimer’s disease, Amyloid-β, Tau, Oxidative stress, Autophagy, Synapses

## Abstract

**Background:**

The homeostasis of metal ions, such as iron, copper, zinc and calcium, in the brain is crucial for maintaining normal physiological functions. Studies have shown that imbalance of these metal ions in the brain is closely related to the onset and progression of Alzheimer’s disease (AD), the most common neurodegenerative disorder in the elderly.

**Main body:**

Erroneous deposition/distribution of the metal ions in different brain regions induces oxidative stress. The metal ions imbalance and oxidative stress together or independently promote amyloid-β (Aβ) overproduction by activating β- or γ-secretases and inhibiting α-secretase, it also causes tau hyperphosphorylation by activating protein kinases, such as glycogen synthase kinase-3β (GSK-3β), cyclin-dependent protein kinase-5 (CDK5), mitogen-activated protein kinases (MAPKs), etc., and inhibiting protein phosphatase 2A (PP2A). The metal ions imbalances can also directly or indirectly disrupt organelles, causing endoplasmic reticulum (ER) stress; mitochondrial and autophagic dysfunctions, which can cause or aggravate Aβ and tau aggregation/accumulation, and impair synaptic functions. Even worse, the metal ions imbalance-induced alterations can reversely exacerbate metal ions misdistribution and deposition. The vicious cycles between metal ions imbalances and Aβ/tau abnormalities will eventually lead to a chronic neurodegeneration and cognitive deficits, such as seen in AD patients.

**Conclusion:**

The metal ions imbalance induces Aβ and tau pathologies by directly or indirectly affecting multiple cellular/subcellular pathways, and the disrupted homeostasis can reversely aggravate the abnormalities of metal ions transportation/deposition. Therefore, adjusting metal balance by supplementing or chelating the metal ions may be potential in ameliorating AD pathologies, which provides new research directions for AD treatment.

## Background

Alzheimer’s disease (AD) is the most common neurodegenerative disorder characterized pathologically by massive extracellular deposition of amyloid-β (Aβ) forming senile plaques and intracellular accumulation of the abnormally modified tau proteins forming neurofibrillary tangles (NFTs) [1, 2].

Aβ is produced from amyloid precursor protein (APP) by cleavage of β- and γ-secretases, in which the β-secretase cleavage is believed to be the rate-limiting step. In the healthy brains, APP is first cleaved by α-secretase to produce an extracellular domain secretory fragment (sAPPa) and a membrane-bound carboxy-terminal fragment. The membrane-bound carboxy-terminal fragment is then cleaved by γ-secretase into small fragments that can be completely degraded, i.e., the non-amyloid pathway. When APP is processed by the amyloid pathway, it is cleaved by β- and γ-secretase to produce Aβ of different lengths. Several gene mutations on APP and presenilin (PS1 and PS2, the γ-secretases) have been identified to promote Aβ production. Furthermore, aggregation of Aβ could form oligomers and plaques, and the oligomers are recognized as the more toxic form of Aβ [3, 4].

Tau is a microtubule-associated protein with the normal function in promoting microtubule assembly and maintaining the stability of the microtubules. No gene mutation has been currently detected on tau in AD patients, instead, multiple abnormal posttranslational modifications have been reported to play roles in AD neurodegeneration, such as hyperphosphorylation, SUMOylation, glycosylation, etc. [5–7]. Recent studies suggest that tau hyperphosphorylation may play a dual role in AD neurodegeneration, i.e., tau hyperphosphorylation renders the cells more resistant to acute apoptosis [8–12], while the increasing intracellular tau accumulation induces multiple cellular impairments, including endoplasmic reticulum (ER) stress, deficits of mitophagy and autophagy, deficits of synaptic transmission, etc.*,* and eventually leads to a chronic neurodegeneration [5, 13, 14].

Clinically, AD is manifested as progressive memory loss, cognitive dysfunction, language disorders, and personality changes. Less than 5% of the AD patients is related to dominant gene mutations, including APP and PS1 or PS2. Animal studies suggest that intervention at embryonic stage is beneficial for inducing synaptic plasticity for these pathological gene carriers [15]. The majority AD patients (> 95%) are sporadic onset in which early diagnosis/prediction or intervention of the high-risk factors, such as type 2 diabetes mellitus and hyperhomocysteinemia, may be recommended [16–18]. Ageing is one of the most recognized causes for sporadic AD. As Chinese society is experiencing a fast increase in the elder populations, the number of AD patients in China is rapidly increasing. Currently, there is no effective drug to cure AD, therefore, understanding the pathological factors that can induce or promote AD is important.

The homeostasis of metal ions is essential for maintaining normal functions of the brain. In AD patients, changes in the dynamic balance of the metal ions in the brain are closely related to the Aβ deposition and tau hyperphosphorylation/accumulation, suggesting a crucial role of the metal irons in the pathogenesis of AD. As both increase and decrease and as well as mis-localization of the metal ions have been observed in AD, several clinical trials by supplementing or chelating or modulating the metal ions have been carried out in AD patients [19].

Iron is the most abundant d-block metal in human body. The iron content in the normal brain is around 0.04 mg/g fresh tissue with the concentration of ~ 720 μM. In the brain, iron is most abundantly detected in the extrapyramidal system, especially in the basal ganglia region; while the iron content is relatively low in cerebral cortex, and it is the lowest in white matter and medulla oblongata [20–22]. An abnormally elevated brain iron is recognized to be the cause of several neurodegenerative diseases, including AD in which the iron accumulation induces cell death, termed as “iron death” [23].

Zinc is the second most abundant d-block metal ion after iron in human body, and it is an essential trace metal for the human. The brain concentration of zinc is estimated at 150 μM, which is ~ 10 times higher than that in serum. In the brain, 80~90% of zinc is tightly bound with proteins to achieve enzymatic activity or structural stability, and over 2800 potential Zn-binding proteins have been identified by proteome. The rest 10~20% of mobile zinc (mZn) are largely stored within synaptic vesicles (> 100 μM) at glutamatergic nerve terminals and it is synaptically released upon neuronal activity, by which it modulates synaptic transmission and multiple biological functions [24, 25]. Both increased and reduced levels of cytoplasmatic zinc have been implicated in AD, suggesting that the intracellular zinc must be tightly regulated to avoid adverse molecular consequences [26].

The copper concentration in human frontal lobe and cerebellum is in the range of 60~110 μM. The highest contents of copper are detected in locus coeruleus and substantia nigra, and copper is also enriched in the hippocampus [27]. Copper is an extremely effective catalyst, which serves as an active component for over 30 enzymes. Complex of copper and Aβ oligomers is able to penetrate the neurons and can trigger oxidative stress within different neuronal sub-compartments. Although copper has been observed to be enriched in the amyloid plaques and NFTs of the AD brains, several copper deficits have also been observed in the AD brains, in which the copper content in cerebral cortex, frontal cortex, amygdala, and hippocampus decreased by up to 50% compared with the control [28, 29].

Calcium is one of the highest metal ions (~ 1200 g) in adult human body. The extracellular concentration of Ca^2+^ is 10^− 3^ M, which is almost ten-thousand times higher than that of the intracellular Ca^2+^ (10^− 7^ M). As a common second messenger, cellular calcium homeostasis plays a pivotal role in regulating many neuronal functions, including neural growth and differentiation, action potential, synaptic plasticity, and learning and memory. In AD experimental models, the intraneuronal calcium concentration is increased [30], and an elevated calcium level generally appear to be toxic to the cells and it triggers subsequent pathological processes of AD.

In the following section, we review the role of metal ions in the pathogenesis of AD, focusing on the role of iron, copper, zinc, and calcium in Aβ and tau pathologies, oxidative stress, autophagic and synaptic deficits.

## Main text

### Role of metal ions in AD-like Aβ and tau pathologies

Erroneous deposition of iron, copper, zinc, or elevation of calcium in different brain regions can promote Aβ overproduction, tau hyperphosphorylation and their aggregation/accumulation. The abnormality of the related metal transporters is a key factor inducing the incorrect distribution of metal ions in the brain.

### Iron

Iron is an essential nutrient but high levels of iron are toxic mainly due to the catalytic generation of destructive hydroxyl radicals. Iron is transferred into the neurons via transferrin (Tf), divalent metal transporter 1 (DMT1), and lactoferrin (Lf), while it is transferred out of neurons by ferroportin (FPN). As a multifunctional iron-binding globular glycoprotein, Lf has high affinity for Fe^3+^. Iron can be transported to tissues and organs with blood circulation by bounding to Tf. Therefore, the dysregulation of DMT1, Lf, Tf and FPN not only affects the distribution but also accumulation of iron in the brain.

An increased iron level is correlated with the amount of Aβ plaques and tau pathologies, and iron increases Aβ toxicity by impeding the ordered aggregation of Aβ [31]. The binding of iron to Aβ or to tau induces Aβ aggregation and tau hyperphosphorylation, leading to formation of senile plaques and NFTs, and as well as the increased neurotoxicity of Aβ and tau [32, 33]. Iron increases APP translation and Aβ42 production/accumulation in the retina with no observed change in secretase levels or cleavage activities [34]. Fe^2+^ ions bound to the N-terminal region of Aβ, which can modify Aβ and generate oxygen radicals to induce damages on the membrane surface [35]. In a human neuroblastoma cell line (SHSY5Y), elevated Fe^3+^ can bind to APP mRNA and promote APP translation, and the Fe^3+^ can activate β-secretase and thus induces Aβ production [36]. It was also shown that huperzine A could attenuate Aβ and tau pathologies in APP/PS1 mice, while feeding the animals with a high iron diet abolished the protective effect of the huperzine A [37]. The increased iron inhibits α-secretase through down-regulating furin [38]. These studies together suggest a detrimental role of iron in AD. Therefore, maintaining iron homeostasis can inhibit APP translation and Aβ overproduction by activating α-secretase [39]. On the other hand, it was reported that iron could promote APP translation to attenuate the toxicities of lead (Pb) [40], suggesting a protective role of iron in APP metabolism. In addition to the effects of iron on APP as mentioned above, APP could reversely affect iron, i.e., to stabilize the iron exporter FPN1, and the heavy subunit of the iron-storage protein, ferritin; by which the elevated APP can regulate metabolisms of brain and peripheral iron to attenuate brain iron elevation during aging [41].

Fe^2+^ can promote tau phosphorylation by activating cyclin-dependent kinases-5 (CDK5) and glycogen synthase kinase-3β (GSK-3β), while the iron chelator deferoxamine can reduce the degree of iron-induced tau hyperphosphorylation in the mouse brain [42]. Iron-mediated tau hyperphosphorylation may be caused by the activation of extracellular regulated protein kinase1/2 (ERK1/2) and mitogen-activated protein kinase (MAPK) pathway [43, 44]. On the other hand, it was also observed that treatment of the cultured hippocampal neuron with Fe^3+^ decreases tau phosphorylation, which correlates with a decreased activity of CDK5 [45]. The differences of the experimental systems used in the studies may at least partially contribute to the observed discrepancy. A recent stduy has also shown that tau protein is reqiured to mediate iron export, which can prevent ferroptotic damage after ischemic stroke [46].

### Copper

In AD patients, an increased copper depositing has been detected and it can interact with Aβ and tau proteins to promote the pathological aggregation and deposition of Aβ and tau proteins.

Copper ions can bind to Aβ and induce oligomer formation, and eventually lead to Aβ aggregation. Cu^2+^-stabilized Aβ1–42 interacts with the lipid bilayer and thus increases the permeability of membrane [47]. However, nitration of Aβ1–42 can dramatically inhibit the Cu^2+^-induced Aβ1–42 oligomerization and neurotoxicity in vitro [48]. Furthermore, Cu^2+^ significantly affects the amyloid cascade, by which it interacts with APP through a Cu^2+^-binding domain [49]. Copper chelator can inhibit the activity of β-secretase [50]. In a preclinical AD model, small amounts of copper in drinking water can induce Aβ accumulation and significantly retard the learning ability [51]. Interestingly, it was also reported that the complex formed by Aβ, monoamine neurotransmitters and Cu^2+^ can mediate Cu^2+^ translocation, by which it reduces Aβ toxicity [52].

Excess copper can promote tau hyperphosphorylation and the copper chelating agents attenuate tau phosphorylation in human neuroblastoma cells. Suppressed levels of copper in plasma and brain resulted in a marked attenuation of tau phosphorylation in transgenic mouse expressing human tau [53]. The copper-responsive transcription factor, Sp1, can bind to the promoter of tau gene, which also links copper to the transcription of tau [54].

### Zinc

Regarding the alterations or role of zinc in AD, large numbers of controversial results have been reported. Some demonstrated that zinc concentration in senile plaques and neuropils of AD increased 2~3-fold compared with the controls, while others showed a decreased zinc concentration in hippocampus or amygdala of AD patients. The reasons for the discrepancy are not very clear, different experimental systems used for the study could be at least one of them. These evidences strongly suggest that zinc is required for normal neural functions while excessive or misdirected zinc is harmful to the neural cells.

By analyzed 48 independent plaques in the hippocampus, James et al. demonstrated that the areal concentrations (ng cm^− 2^) of iron, copper and zinc were significantly higher in plaques than in the surrounding neutrophils in APP/PS1 mice. Interestingly, only the level of zinc in the plaques remained elevated after adjustment of tissue density [55]. In addition to increasing protein deposition, direct binding of zinc to Aβ also reduces the solubility of Aβ and thus exacerbates damage to neurons by increasing Aβ aggregation [56]. Immediately after addition of Aβ1–40, zinc ions can block the cation channel on the surface of the membrane, but subsequent Aβ fiber formation fragments the membrane that cannot be stopped by zinc [57]. Zinc ions are associated with APP processing, and interference in the hydrolysis of the APP by zinc results in abnormal cleavage of APP and deposition of Aβ, possibly owing to the decreased α-secretase cleavage of the zinc-bound APP or the increased Aβ production from APP in the presence of zinc. Additionally, a disintegrin and metalloprotease 10 (ADAM10), whose function can be activated by zinc, can also catalyzes the non-amyloidogenic α-secretase cleavage of the APP [58, 59].

Excessive of zinc also induces tau aggregation and formation of the NFTs. Zinc can directly bind to tau monomers and stimulate the phosphorylation of tau proteins by activate GSK-3β, ERK1/2, and c-Jun N-terminal kinase (JNK) [60, 61]. Zinc also induces protein phosphatase 2A (PP2A) inactivation and tau hyperphosphorylation through Src-dependent pathway, which ultimately leads to a net increase in phosphorylated tau that may exacerbate AD-like tau pathologies. In genetically modified mice with high expression of human tau protein, zinc chelating agent inhibited the activation of Src, thereby reduced the inhibitory phosphorylation of PP2A at Tyr307 with attenuation of tau phosphorylation and aggregation [62, 63].

### Calcium

Intracellular Ca^2+^ dysregulation is an early manifestation of AD. Studies have shown that Ca^2+^ concentrations near Aβ deposits are significantly increased, and the increase of Ca^2+^ likely plays a key role in the cognitive deficits associated with AD [64, 65]. Ca^2+^ elevation can promote Aβ production and the cellular toxicity of Aβ, while the elevation of Aβ in turn contributes to the increased intracellular Ca^2+^. Therefore, a positive feedback loop may exist between Ca^2+^ and Aβ, which may exacerbate the neurodegeneration and cognitive deficits in AD patients [66].

Aβ can promote the opening of voltage-dependent calcium channels, which in turn increases the intracellular concentration of Ca^2+^ [67]. An increased intracellular Ca^2+^ concentration can promote the overexpression of L-type calcium channel subtype (Cav1.2) in the hippocampal cell membranes in models of AD, which further promote the influx of Ca^2+^ [68]. Majority reports suggest that the intracellular calcium overload can promote Aβ production and aggregation. For instance, the elevated cytoplasmic Ca^2+^ by inhibiting of sarcoplasmic/endoplasmic reticulum (S/ER) calcium ATPase (SERCA) or liberating Ca^2+^ release via the ryanodine receptor (RyR) can robustly activate β-secretase and thus increase Aβ production [69]. Therefore, inhibition of Ca^2+^ influx can reduce the neurotoxicity of Aβ oligomers, levels of insoluble Aβ1–40 and Aβ1–42 in the hippocampus of AD transgenic mice [70]. Studies also demonstrated that Ca^2+^ ions actively participate in Aβ-promoted membrane damage, i.e., Ca^2+^ ions inhibit Aβ-mediated membrane poration but enhance membrane fragmentation by lipid loss due to fiber growth on the membrane surface [71].

Given that many kinases can be activated by Ca^2+^, dysregulation of Ca^2+^ homeostasis, such as seen during ER stress, can increase tau phosphorylation [14, 72, 73]. Conversely, intracellular tau accumulating can also induce ER stress and cause Ca^2+^ overload, the latter induces dephosphorylation of Ca^2+^-calmodulin-dependent protein kinase IV (CaMKIV) and cAMP-responsive element binding protein (CREB) by activating calcineurin [5]; the Ca^2+^ overload can also activate JAK2-STAT1 signaling, and the upregulated STAT1 directly binds to the specific GAS elements of N-methyl-D-aspartate receptors (NMDARs) and thus inhibits the transcription of NMDARs [14]; the cleaved tau-induced STAT1 elevation also activates BACE1 to promote Aβ production [74]; all of which reveal new mechanisms underlying tau-induced synapse impairments and cognitive deficits.

### Role of metal ions in oxidative stress

Oxidative stress is a state of cellular damage caused by free radicals because of the insufficient functioning of the antioxidant system [75]. Under physiological conditions, the peroxidation-antioxidant system is in equilibrium. When this balance is disturbed, the body responds to oxidative stress by producing free radicals, including reactive oxygen species (ROS), such as •O_2_, H_2_O_2_, and •OH, and reactive nitrogen species (RNS), such as •ONOO and •NO.

Neurons in the brain are extremely sensitive to free radicals. DNA damage, protein oxidation, lipid peroxidation, and production of advanced glycosylation end products (AGEs) in the AD brains are usually related to free radical attacks and metal imbalances [76, 77]. Oxidative stress has been wildly detected in AD patients and the animal models, and the imbalance of metal ions, such as iron, copper, zinc, and calcium, can cause oxidative stress, tau hyperphosphorylation, Aβ deposition, cross-linking of nerve fibers and nerve cell damages, which are closely related to the pathogenesis of AD.

### Iron

The reversible transition of iron from Fe^2+^ to Fe^3+^ catalyzes the electron transfer reaction. However, excessive redox reactions can disrupt the transition. The iron content in the brain is tightly regulated. When the concentration of iron in the brain is too high, oxidative stress occurs through the Haber-Weiss and Fenton reaction that directly generates ROS. The overproduction of ROS subsequently causes lipid peroxidation of the neuronal membrane, DNA damage, and neuron impairment in the brain [78, 79].

Oxidative stress has been observed in mild cognitive impairment with a correlated elevation of iron in glial cells and cerebellum. The glia act as immune cells in the brain and participate in maintaining homeostasis and multiple brain functions. Though the role of cerebellum in AD has not been extensively studied, the increased iron and free radical generation had been detected in both glial cells and cerebellum in the early stage of AD [80], and in cerebellum, loss of Purkinje cells and synaptic alterations in the mossy fibers, granule cell dendrites, parallel fibers and Purkinje cell dendrites with substantial loss of dendritic spines had been shown. High levels of iron were precipitated with Aβ plaques, and the iron can promote toxic Aβ oligomer formation with production of ROS, leading to mitochondrial dysfunction and cell death [81].

### Copper

Copper is a cofactor for Cu/Zn-superoxide dismutase and plays a key role in scavenging ROS. An imbalanced homeostasis of copper can induce oxidative stress and cause ROS overproduction by Fenton and Haber-Weiss reactions [82]. Interferes with the bidirectional and copper-dependent communication between neurons and astrocytes could eventually lead to various brain diseases.

APP and Aβ have copper binding sites, and their interaction with copper can produce ROS, including •OH, which can cause oxidative damage to Aβ itself and the surrounding proteins and lipids. Moreover, elevated copper reduces the level of GSH, a substrate for enzymes that remove ROS, and enhances the production and cytotoxic effects of ROS [83]. The expression of copper-dependent enzymes, such as superoxide dismutase 1 (SOD1) and antioxidant protein 1 (ATOX1), was markedly reduced in multiple microarray studies of AD patients, reinforcing the lack of copper [84]. In addition to the antioxidant role, SOD1 also has anti-inflammatory functions. Therefore, reduced expression of SOD1 can exacerbate ROS accumulation and the chronic neuroinflammation [85].

### Zinc

Although zinc is recognized to be a redox-inert metal ion, there are still evidences showing an active involvement of zinc in redox metabolism. In the brain, metallothionein 3 (MT3) is one of the major players in maintain the homeostasis of zinc, which is chelated as metal-thiolate clusters. MT3 regulates zinc in a copper-related manner, and the zinc in MT3 can exchange with copper in the Aβ-Cu complex and inhibit the oxidative damage caused by the Aβ-Cu complex, Zn^2+^ can also protect sulfhydryl groups from oxidation in the cells [86]. Therefore, downregulation of zinc or MT3 as observed in the AD neurons can contribute to the oxidative damages.

Under pathological conditions, excessive zinc is released from presynaptic neurons and astrocytes, which causes NADPH-oxidase activation and ROS production in neurons, and microglial activation, and eventually exacerbates neuronal death [87]. Furthermore, Zn^2+^ is an inhibitor of many enzymes, therefore, excessive zinc can cause multiple metabolic abnormalities by deregulating related enzyme activities. The mitochondrial respiratory chain is particularly sensitive to Zn^2+^, and elevated Zn^2+^ in mitochondria promotes ROS production. Excessive zinc, which can be caused by an increased release from metalloproteins, can induce Aβ production and deposition, and thereby triggering a cascade reaction. Therefore, the oxidative and nitrosative stresses can cause an uncontrolled zinc elevation and Aβ deposition, while zinc and Aβ accumulation can conversely lead to oxidative stress and cytotoxicity, forming a vicious cycle.

### Calcium

Calcium-mediated oxidative stress is largely inseparable from iron. Recent studies show that iron-mediated calcium signaling leads to the downstream activation of a kinase cascade involved in synaptic plasticity. Under physiological conditions, iron-mediated production of ROS promotes a normal calcium-dependent signaling pathway, while excessive iron promotes oxidative stress, leading to an unrestricted increase in calcium signaling that impairs mitochondrial function and other downstream targets [88, 89]. Activation of microglia and astrocytes in the brain of AD patients can induce ROS and RNS production with dose-dependent increases of glutamate release and calcium entry, leading to neuron death.

### Role of metal ions in autophagy

Autophagy, a highly conservative proteolysis system driven by the lysosome, can remove dysfunctional organelles and misfolded proteins and thus plays an important role in coping with various adverse environmental stresses and maintain cell vitality [90, 91]. An insufficient autophagy can cause intracellular protein accumulation and thus impair the cellular functions, while excessive autophagy can also destroy the cell microenvironment and cause cell damages. Though both insufficient autophagy and excessive autophagy have been detected in the AD brains, an insufficient autophagy may play a more important role in the accumulation of the misfolded proteins during AD [92, 93], such as tau and Aβ accumulation. Interestingly, a recent study showed that intracellular accumulation of tau proteins could in turn aggravate autophagy deficits by disrupting IST1-regulated ESCRT-III complex formation and autophagosome-autolysosome fusion, which formed a vicious cycle between tau accumulation and autophagy deficit in AD and the related tauopathie [13]. Therefore, autophagy should be a promising therapeutic target for AD, and metal ions change the autophagy processes.

The activity of ubiquitin proteasome system (UPS) is decreased in the AD brains, and the UPS activity is closely intertwined with autophagy functions. Moreover, the copper, Fe and zinc ions can bind to UPS components, e.g., the proteasome and ubiquitin can and thus modify their activity [94].

### Iron

The increased iron accumulation is commonly seen in the brains of AD patients. Uptake of iron components and lysosomal accumulation of iron-related lipofuscin over time induces autophagy deficits. The iron-rich autolysosomes can produce more ROS that can cause damage to autolysosomal membranes [95]. Ferritin is an intracellular protein that stores iron; therefore, autophagy deficit-related ferritin elevation plays an important role in accumulating iron and mediating the iron toxicities in the AD [96, 97]. Additionally, an increasing iron in the cytoplasm will saturate ferritin and thus prevent ferritin from entering the autolysosome which exacerbates iron accumulation. On the other hand, a rapidly increased iron can activate AMP-activated protein kinase (AMPK)/mammalian target of rapamycin (mTOR) and promote lysosomal degradation of Aβ, which suggests a protective role of a rapidly increased iron in Aβ removal. It is reported that iron exposure in the neonatal period has long-lasting harmful effects on the UPS which can induce memory impairment [98]. The proteasome inhibitor lactacystin causes a marked increase in labile iron and the aggregation of ubiquitin-conjugated proteins prior to cell injury and death in vitro [99].

### Copper

In vitro experiments have shown that excessive addition of copper to the cell culture medium can induce autophagy, and the autophagy here is recognized as a cellular defense mechanism that can reduce cell death [100]. The elevation of copper has been observed in lysosomes, the organelles involved in autophagy [101]. In the redox cycle of metal ions, elevation of copper promotes autophagy and apoptosis of glioma cells by reactive oxygen species and JNK activation [102, 103]. The activity of proteasome can be inhibited by copper at mM level and copper-chelator complexes can inhibit it [104]. And it is reported Cu^2+^ ions inhibited interaction of nerve growth factor (NGF)1–14 and ubiquitin [105] .

### Zinc

Zinc plays a role in the regulation of both basal and stress-induced autophagy. When the concentration of Zn^2+^ is high, the level of autophagy will be increased [106]. Zinc promotes autophagy with the mechanisms involving ERK1/2 activation, which can phosphorylate beclin-1 and thus facilitate the beclin1-PI3K complex formation during autophagic process; zinc can also promote degradation of mTOR, a negative regulator of autophagy, and thus lead to cell autophagy [107]. It was also reported that zinc oxide nanoparticles-induced autophagy may be associated with oxidative stress and the inflammatory process in primary astrocyte cultures [108]. Addition of increasing Zn^2+^ may interact with specific regions of ubiquitin and promote protein-protein contacts [109]. And zinc caused UPS impairment resulting in α-synuclein aggregation subsequently leading to dopaminergic neurodegeneration [110]. In PC12 cell, zinc-induced autophagosome formation facilitates cell survival [111]. These data suggest that the increased zinc in the AD brain may be potentially protective in removal of stress-induced abnormal proteins/organelles by autophagy, although the pathological role of the increased zinc in AD-like tau and Aβ pathologies and protective role of zinc chelator were also reported [112].

### Calcium

In physiological conditions, the extracellular calcium concentration is thousands-fold higher than that of the intracellular calcium. In the face of metabolic pressures, such as hypoxia and nutrient deprivation, transient receptor potential mucolipin 1 (TRPML1) mediates outflow and increases the concentration of Ca^2+^ in the cytoplasm [113]. With increase of intracellular Ca^2+^, autophagic vesicles is accumulated and autophagy is stimulated, the mechanisms involve Ca^2+^/calmodulin-dependent protein kinase kinase β (CaMKKβ)/AMPK activation and mTORC1 inhibition [114, 115]. Application of BAPTA-AM, a chelator of cytoplasmic Ca^2+^, can inhibit the accumulation of autophagic vesicles, which confirms the role of Ca^2+^ in stimulating the autophagy [116].

### Role of metal ions in synapses

Synapse impairment is an early pathological feature of AD, which is positively correlated with cognitive dysfunction in AD [117]. The homeostasis of metal ions is critical for maintaining synaptic function.

### Iron

Iron is closely related to synaptic functions. It has been reported that the dietary iron is required for synapse formation and iron deficiency reduces synapse formation in the drosophila clock circuit. Iron mediates NMDAR-dependent stimulation of calcium-induced pathways and hippocampal synaptic plasticity. Non-transferrin-bound iron (NTBI) contributes to the “oxidative tone” which is important for both basal synaptic transmission and long-term potentiation [118]. In the presence of elevated iron, increased synaptic activity can cause iron overload which concurs to the cytotoxic effects, as seen in early stage of AD patients [119]. On the other hand, long-term excessive iron exposure induces learning and memory deficits in mice with widespread molecular alterations including synapse- and memory-associated proteins [120].

### Copper

Copper is an essential component of neuronal transmission. Upon synaptic depolarization, copper release at micromolar concentrations from copper-containing vesicles into the synaptic cleft modulates synaptic functions [121, 122]. Once released onto the synaptic cleft, copper can act post-synaptically as a high-affinity blocker of NMDAR and glutamatergic ɑ-amino-3-hydroxy-5-methyl-4-isoxazolepropionic acid receptor (AMPAR), thereby producing a marked inhibition of glutamate-mediated neurotransmission [123]. Copper can also control, directly or indirectly, the activity of γ-aminobutyric acid (GABA) and P2X receptors and thus affect neurotransmission and neuronal excitability. Copper can also modulate synaptic vesicles trafficking and protein interactions, while the neurotransmission can in turn affect copper trafficking and delivery in neuronal cells.

As mentioned above, copper can promote Aβ production and tau hyperphosphorylation. In turn, the aggregating vulnerable proteins, such as APP, prion protein, α-synuclein, show copper-binding domains. Therefore, these proteins may act as copper buffers at synapses and participate in the interplay between copper and the neurotransmitters receptors. Mutations of copper pump ATP7A are responsible for disorders with a prominent neurodegenerative component, suggesting that ATP7A may play a pivotal role in the release of copper at synapses. The involvement of copper in synaptic transmission may open new insights for therapeutic interventions of AD.

### Zinc

Among different types of metal ions, zinc is the most extensively studied and recognized one to be involved in regulating synaptic functions. Zinc ion is a potent allosteric inhibitor of NMDA receptors. By its mobile form accounting for 10~20% of total zinc, the mZn presents in the excitatory presynaptic vesicles with the concentration > 100 μM, while the inhibitory neurons have been recognized as no-zinc cells. Excessive intracellular accumulation of Zn^2+^ induces toxicities through multiple mechanisms, such as increasing ROS production through damaging mitochondrial functions, as often seen after the acute phase of transient ischemia or epilepsy.

Among the three major routes of divalent cation entry, i.e., the NMDA channels, the voltage-sensitive Ca^2+^ channels (VSCCs), and the Ca^2+^-permeable AMPA/kainate (Ca-A/K) channels, the Ca-A/K channels show the highest permeability to exogenously applied Zn^2+^. In a model of trans-synaptic Zn^2+^ movement occurring under conditions of oxygen and glucose deprivation (OGD), it was observed that blockage of NMDAR or VSCC by using MK-801 or Gd^3+^ increased Zn^2+^ accumulation, whereas the blocking Ca-A/K channels by using 1-naphthyl acetyl spermine (NAS) or application of the extracellular Zn^2+^ chelator (Ca^2+^ EDTA) decreased Zn^2+^ accumulation in the pyramidal neurons of hippocampal CA1 and CA3 subsets. Simultaneously, significant neuron death was shown in the presence of MK-801 and Gd^3+^, whereas the injury was attenuated by NAS or Ca^2+^ EDTA. These data suggest a pivotal role of Ca-A/K channel, a route for rapid and direct Zn^2+^ entry, in zinc accumulation and the neuronal toxicities. Similar to that seen in OGD, downregulation of GluR2 in the dendrites of pyramidal neurons progressively increases the number of Ca-A/K channels with an increased Zn^2+^ inflow and an increased neuron loss during aging and AD [124].

Intriguingly, the Ca-A/K channel is highly expressed in hippocampal pyramidal neurons, basal forebrain acetyl-cholinergic neurons, and the forebrain somatostatin neurons, while these neurons are prominently injured in AD patients. This evidence imply that proper zinc entry must be protective during AD. The synaptic activity could improve neuronal resistance to apoptosis via synaptically released zinc, whereas lowering Zn^2+^ concentration by using intracellular chelators can cause apoptotic neuronal death [125]. In the central nervous system, the brain-derived neurotrophic factor (BDNF) can modulate critically the neuronal activities, covering from neuronal differentiation and survival to synaptogenesis and activity-dependent synaptic plasticity [126, 127]. The production and maturation of BDNF in the brain is regulated by Cu^2+^-dependent metalloproteinases. In SHSY5Y cells, Cu^2+^ treatment decreased pro-BDNF level in cells and increased pro- and mature BDNF levels in the medium [128] with a strong decrease of the proliferative activity of both cleaved BDNF (1–12) and the full length protein [129]. Application of zinc chelator induced reduction of BDNF level, synaptic plasticity-related proteins and dendritic spine density in vivo, which further confirm that appropriate amount of Zn^2+^ is essential in brain development and the synaptic functions [130].

At the cellular level, mZn is loaded onto the presynaptic vesicles by zinc transport protein ZnT3, by which zinc plays a role in modulating neurotransmission and plasticity of glutamatergic neurons. The mice with ZnT3 knock out exhibit learning and memory deficits only at 6-months but not at 3-months of age. These age-dependent cognitive deficits are associated with significantly decreased levels of key hippocampal proteins involved in learning and memory. As aggregation of extracellular Aβ may trap this synaptic pool of Zn^2+^, ZnT3 knock out may represent a phenocopy for the synaptic and memory deficits of AD. Interestingly, this ZnT3 deletion mice also showed a reduced synaptic localization of Aβ oligomerization compared to that in wild-type mice [131], which implies a functional role of Aβ oligomer in the synapses.

### Calcium

In AD, increased levels of Ca^2+^ in dendrites and dendritic spines have important effects on synaptic dysfunction. Calcineurin is an important enzyme that mediates the signaling pathway of Ca^2+^ in the central nervous system. Calcineurin can work cooperatively with Aβ or tau and contribute to the loss of dendritic spines and synapses, leading to cognitive deficit in mouse models of AD, thus use of calcineurin inhibitors can reverse or improve these impairments [5, 132].

Mitochondria are especially abundant in synapses, where they provide energy for calcium homeostasis and synaptic functions. The synaptic mitochondria are more sensitive to calcium overload damage than non-synaptic mitochondria [133]. Cytoplasmic Ca^2+^ can flow into the mitochondria and activate the calcium-dependent mitochondrial matrix dehydrogenase to produce ATP. However, the cytoplasmic Ca^2+^ overload causes excessive Ca^2+^ influx into the mitochondria. The mitochondrial Ca^2+^ overload induces ROS overproduction, and thus results in mitochondrial outer membrane permeabilization (MOMP) and the subsequent release of pro-apoptotic factors into the cytoplasm. The pro-apoptotic factors, including cytochrome C and apoptosis inducing factor, activate caspases that induce apoptosis [134]. During long-term potentiation (LTP), microtubule associated protein 1B (MAP1B) phosphorylation and local concentrations of Ca^2+^-calmodulin-dependent kinase II (CaMKII) were increased [135]. CaMKII is responsible for phosphorylating MAP2, which enhances synaptic response [136]. The human tau accumulation impairs synapse and memory by activating Ca^2+^-dependent calcineurin and suppressing nuclear CaMKIV/CREB signaling, which reveals a new mechanism underlying the human tau-induced synaptic toxicity [5].

**Fig. 1:**
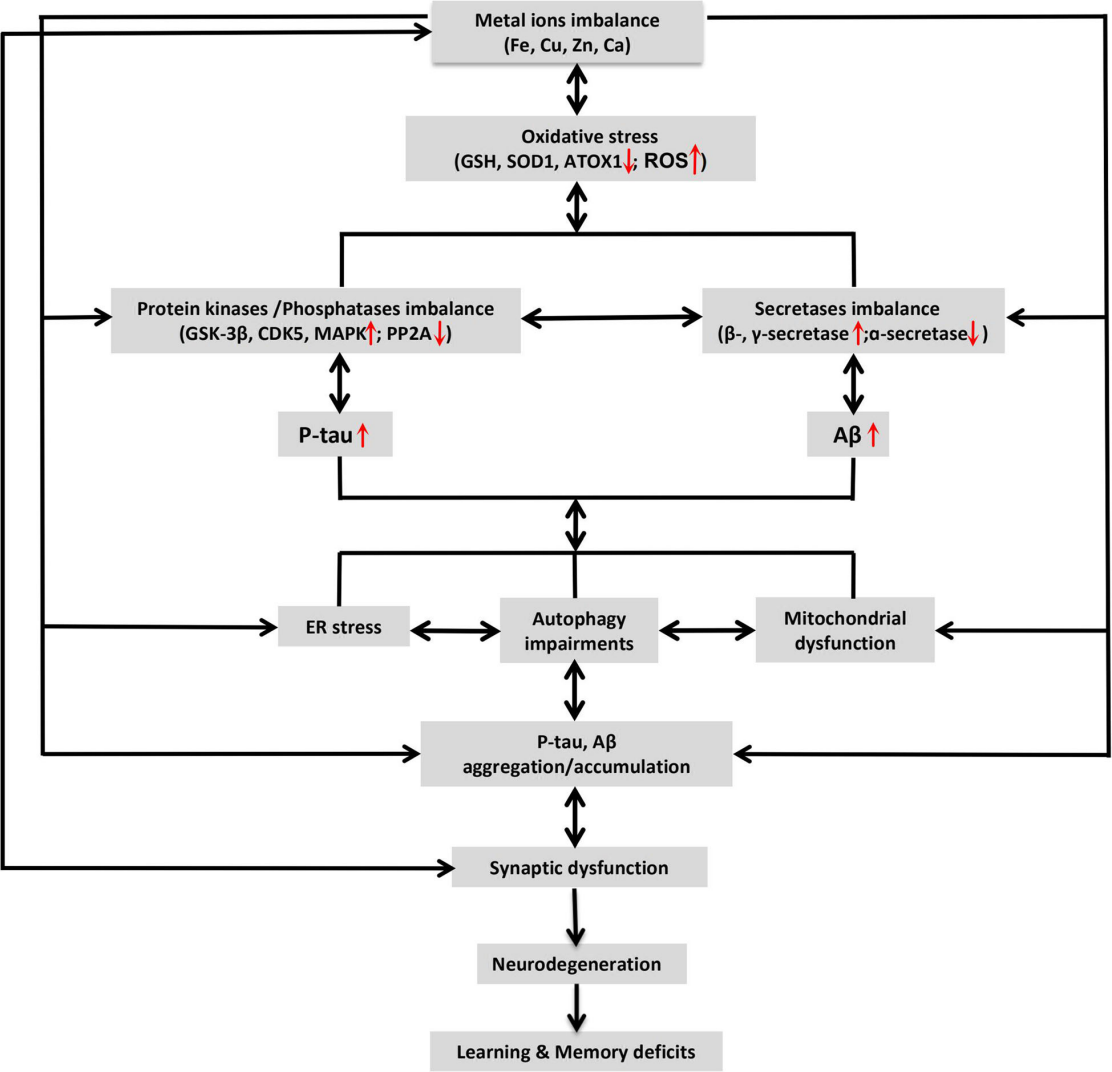
Metal ions imbalance in Alzheimer-like neurodegeneration and cognitive deficits. The metal ions (Fe, Cu, Zn and Ca) imbalance induces oxidative stress demonstrated by the reduced level/activity of GSH, SOD1 and ATOX1 and an increased level of ROS. Oxidative stress can induce tau hyperphosphorylation by activating protein kinases (such as GSK-3β, CDK5, MAPK, etc.) and/or inhibiting PP2A; it can also promote Aβ overproduction by activating β- and γ-secretases and/or inhibiting α-secretase. Together with the imbalanced metal ions and oxidative stress, or independently, the hyperphosphorylated tau (p-tau) and overproduced Aβ could induce ER stress, mitochondrial dysfunction, and autophagic impairments, leading to p-tau and Aβ aggregation and accumulation. Again, together with p-tau/Aβ accumulation, autophagic/mitochondrial deficits, ER stress and oxidative stress, or independently, the imbalanced metal ions can induce synapse damages, which causes synaptic dysfunction, neurodegeneration, and eventually learning and memory deficits. As indicated in the figure by the double-sided arrows, many of these pathological processes occur in a bi-directional way and thus form a vicious cycle during the age-dependent chronic neurodegeneration, such as seen in AD

To help the readers quickly understand the key points, we summarized how metal ions (Fe, Cu, Zn and Ca) are involved in AD-like neurodegeneration and memory deficits, mainly from their effects on oxidative stress, Aβ and tau pathologies, autophagic imbalance, and synaptic impairment (Fig. 1).

## Conclusions

The homeostasis of metal ions is critical for the physiological functions of the brain. In AD patients or the animal models, the imbalanced metal ions and their transporters have been widely observed. The deposition of metal ions in different brain regions impairs mitochondrial functions and thus causes oxidative stress, which can result in cascade pathological reactions. By modulating specific protein kinases and/or phosphatases or β-, γ-, α-secretases or inducing oxidative stress, the elevated or imbalanced metal ions can induce or exacerbate Aβ overproduction, tau hyperphosphorylation and Aβ/tau aggregation. Metal ions can also promote or inhibit autophagy by acting on autophagy-related proteins and thus causes autophagy impairments, leading to/or aggravating abnormal intracellular protein accumulation. The synaptic release of certain metal ions is essential for normal synaptic plasticity and the functions, whereas abnormal distribution or metabolism of metal ions induces synaptic dysfunction during the progression of AD, with the mechanisms closely related to the mitochondria in the synapses (Fig. 1).

Although both increase and decrease of the metal ions have been observed in AD, the increased ions accumulation/overload are more commonly seen. Therefore, application of metal ions chelators has been widely studied for ameliorating pathologies and the cognitive functions in AD. An effective metal chelator should have the following features: (i) able to pass through the blood-brain barrier (BBB), (ii) specific for a single metal ion, and (iii) no interference to the normal metabolism of the metal ions. Therapeutic chelators must take into account the necessary coordination to select suitable candidates and the possible success in clinical trials. Therefore, many attempts are made to find metal chelators as drug candidates in recent years, but few chelators have been rationally designed to create safe and effective drugs [137]. As severe deficiency of metal ions has also been observed in the AD brains and it may also contribute to AD neurodegeneration, therefore, supplement of specific metal ions should be considered to these patients. In addition, modulating the cellular localization of the metal ions is also an important approach for developing AD therapies against metal ions.

Currently, it is still not clear how exactly the concentrations and the distributions of the above-mentioned metal ions are changed during aging and AD. It is also elusive how the brain region-, neural cell-, and sub-organelle-specific distribution of the metal ions are normally regulated or maintained in the brain; and how these regulating/maintaining systems are changed during aging and AD. In future studies, more compelling data on these aspects will be meaningful for designing and developing specific interventions.

## Data Availability

Not applicable.

## Abbreviations

AD: Alzheimer’s disease
ADAM: A disintegrin and metalloprotease
AGEs: Advanced glycosylation end products
AMPAR: ɑ-amino-3-hydroxy-5-methyl-4-isoxazolepropionic acid receptor
AMPK: AMP-activated protein kinase
Aβ: amyloid-β
APP: Amyloid precursor protein
ATOX1: Antioxidant Protein 1
BDNF: Neurotrophin brain-derived neurotrophic factor
BBB: Blood-brain barrier
Ca-A/K: Ca^2+^-permeable AMPA/kainate
CaMK: Ca^2^-calmodulin-dependent protein kinase
CaMKKβ: Ca^2+^/calmodulin-dependent protein kinase kinase β
CaMKII: Ca^2+^-calmodulin-dependent kinase II
Cav1.2: L-type calcium channel subtype
CDK5: Cyclin-dependent kinase-5
CREB: cAMP-responsive element binding protein
DMT1: Divalent metal transporter 1
ER: Endoplasmic reticulum
ERK1/2: Extracellular regulated protein kinase 1/2
FPN: Ferroportin
GABA: γ-aminobutyric acid
GAPDH: Glyceraldehyde-3-phosphate dehydrogenase
GSK-3β: Glycogen synthase kinase-3β
JNK: c-Jun N-terminal kinase
Lf: Lactoferrin
LTP: Long-term potentiation
MAPK: Mitogen-activated protein kinase
MAP1B: Microtubule associatedprotein1B
MOMP: Mitochondrial outer membrane permeabilization
MT3: Metallothionein 3
mTOR: mammalian target of rapamycin
NAS: 1-naphthyl acetyl spermine
NFTs: Neurofibrillary tangles
NGF: Nerve growth factor
NMDAR: N-methyl-D-aspartate receptor
NTBI: Non-transferrin-bound iron
OGD: Oxygen and glucose deprivation
PP2A: Protein phosphatase 2A
PS: Presenilin
RNS: Reactive nitrogen species
ROS: Reactive oxygen species
RyR: ryanodine receptor
SERCA: Sarcoplasmic/endoplasmic reticulum calcium ATPase
SOD1: Superoxide dismutase 1
Tf: Transferrin
TRPML1: Transient receptor potential mucolipin 1
UPS: ubiquitin proteasome system
VSCCs: Voltage-sensitive Ca^2+^ channels
